# Rethinking walkability and developing a conceptual definition of active living environments to guide research and practice

**DOI:** 10.1186/s12889-022-12747-3

**Published:** 2022-03-07

**Authors:** Melissa Tobin, Samantha Hajna, Kassia Orychock, Nancy Ross, Megan DeVries, Paul J. Villeneuve, Lawrence D. Frank, Gavin R. McCormack, Rania Wasfi, Madeleine Steinmetz-Wood, Jason Gilliland, Gillian L. Booth, Meghan Winters, Yan Kestens, Kevin Manaugh, Daniel Rainham, Lise Gauvin, Michael J. Widener, Nazeem Muhajarine, Hui Luan, Daniel Fuller

**Affiliations:** 1 School of Human Kinetics and Recreation, Memorial University of Newfoundland, St. John’s, Newfoundland and Labrador, A1C 5S7 Canada; 2 MRC Epidemiology Unit, Institute of Metabolic Science, School of Clinical Medicine, University of Cambridge, Cambridge, UK; 3 Department of Geography, McGill University, Montreal, QC Canada; 4 School of Mathematics and Statistics, Carleton University, Ottawa, ON Canada; 5 School of Population and Public Health, University of British Columbia, Vancouver, BC Canada; 6 Cumming School of Medicine, University of Calgary, Calgary, AB Canada; 7 Department of Geography, Western University, London, ON Canada; 8 Department of Medicine, University of Toronto, Toronto, ON Canada; 9 Faculty of Health Sciences, Simon Fraser University, Vancouver, BC Canada; 10 École de Santé Publique de L’Université de Montréal (ESPUM), Montréal, Québec Canada; 11 School of Health and Human Performance, Faculty of Health, Dalhousie University, Halifax, NS Canada; 12 Centre de Recherche du Centre Hospitalier de L’Université de Montréal (CRCHUM), Montréal, Québec Canada; 13 Department of Geography and Planning, University of Toronto - St. George, Toronto, Canada; 14 Department of Community Health and Epidemiology, Faculty of Medicine, University of Saskatchewan, Saskatoon, Canada; 15 Department of Geography, College of Arts and Science, University of Oregon, Eugene, OR USA

**Keywords:** Active transport, Built environment, Health, Natural environment, Neighbourhood, Social environment, Sustainable, Walkability

## Abstract

**Background:**

Walkability is a popular term used to describe aspects of the built and social environment that have important population-level impacts on physical activity, energy balance, and health. Although the term is widely used by researchers, practitioners, and the general public, and multiple operational definitions and walkability measurement tools exist, there are is no agreed-upon conceptual definition of walkability.

**Method:**

To address this gap, researchers from Memorial University of Newfoundland hosted “The Future of Walkability Measures Workshop” in association with researchers from the Canadian Urban Environmental Health Research Consortium (CANUE) in November 2017. During the workshop, trainees, researchers, and practitioners worked together in small groups to iteratively develop and reach consensus about a conceptual definition and name for walkability. The objective of this paper was to discuss and propose a conceptual definition of walkability and related concepts.

**Results:**

In discussions during the workshop, it became clear that the term walkability leads to a narrow conception of the environmental features associated with health as it inherently focuses on walking. As a result, we suggest that the term Active Living Environments, as has been previously proposed in the literature, are more appropriate. We define Active Living Environments (ALEs) as the emergent natural, built, and social properties of neighbourhoods that promote physical activity and health and allow for equitable access to health-enhancing resources.

**Conclusions:**

We believe that this broader conceptualization allows for a more comprehensive understanding of how built, natural, and social environments can contribute to improved health for all members of the population.

## Background

Walkability is a common term used in both research and practice in the fields of urban planning, geography, and public health. However, only a small amount of work has focused on developing a conceptual definition of walkability. The term has been used in the academic literature since the late 1990s when researchers began to examine how neighbourhood designs were associated with travel behavior, physical activity, and obesity [1]. Since this time the academic community and working professionals have used a variety of different definitions to understand and measure walkability [2]. The wide-ranging definitions of walkability have not been used consistently across fields and thus have led to confusion and misuse of the term [1]. There is a growing need for a consensus definition of walkability that provides a foundation for conceptualizing and measuring the characteristics of neighbourhood environments that may promote active living and health [3]. In this paper, we report on the outcomes of a pan-Canadian workshop held in St-John’s, Newfoundland in conjunction with members of the Canadian Urban Environmental Health Research Consortium (CANUE) in November 2017. The workshop aimed to develop a consensus around a conceptual definition of walkability and discuss the future of measurement.

Walkability is one component of an overarching set of features about neighbourhoods, but there are environmental features that may be associated with health outcomes that this term excludes. To date, there is no agreed-upon conceptual definition of walkability, as noted by many variations in the literature [4–12]. Grant (2013) defines walkability as an “excellent shorthand for good urban design” [13]. Wikipedia defines walkability as “a measure of how friendly an area is to walking” [9]. Wang and Yang in their review and bibliometric analysis define walkability as “the extent to which the built environment is friendly to people who walk, which benefits the health of residents and increases the liveability of cities. [14]” Each of these definitions is likely correct in part but none is broad enough to encompass all relevant environmental features that may cause active living and do not provide guidance for measurement. We argue that previously proposed conceptual definitions are often too vague [7, 9] or are singularly based on disciplinary perspectives [15]. Definitions often fail to underscore that the extent to which an area is walkable is only one dimension of neighbourhoods [8, 9]. Characteristics that contribute to a “walkable” neighbourhood can also support other physical activities (e.g., recreational strolling, cycling, skateboarding) and health-related behaviours (e.g., socializing, healthy eating). A useful conceptual definition should build on theoretical concepts rather than solely focusing on operational measurement (e.g., the variables to include in a measure) [16].

We identified two papers that proposed revised definitions of walkability in the literature. First, Gauvin et al. (2005) proposed the concept of “Neighbourhood Active Living Potential (NALP)” to extend the concept of walkability, which comprised three underlying dimensions (i.e., activity friendliness, safety, and density of destinations). They stated that NALP can be understood as those “aspects of the neighbourhood that regulate the likelihood of active living in individuals and populations” [6]. To measure this construct, the authors proposed the use of a systematic social observation [17] grid involving an 18-item checklist to be completed by trained personnel directly observing neighbourhoods. More recently, Hajna et al. (2017) suggested using a concept separate from walkability, namely “neighbourhood physical activity environments”, in addition to or instead of the term “walkability”, as it does not restrict the potential effects of environments to promoting a single type of physical activity (i.e. walking) and it does not imply that a universal causal association exists between environments and walking [4]. Both definitions include the notion of neighbourhoods, which in and of itself poses challenges [18]. It is not our objective to discuss challenges with defining exposure boundaries (e.g., neighbourhood, activity space). As such, we will use the neighbourhood to generally mean the concept of spatial exposure [19]. Despite the acknowledged limitations associated with the notion of walkability, neither alternative concept has been widely used in the literature nor in practice.

Often the operational measures of walkability go well beyond the strict concept of what contributes to ‘walking’ per se, but the term walkability continues to be the most used. There are a number of variables that researchers commonly draw upon to operationalize the concept of walkability [4–12]. These are summarized below, with examples of common operational characteristics of walkability from the literature. It is not our intent to systematically summarize the literature but rather to provide a general overview.

Ewing and Cervero (2010) proposed the 5 D’s to measure walkability: density, diversity, design, destination accessibility, and distance to transit [10]. Frank developed and applied a parcel level walkability index in 1995 [20] comprised of measures of proximity (mixed land use and net residential density). The availability of GIS-enabled the creation of observation-specific buffers, connectivity (e.g., intersection density) and site design (e.g., retail floor space ratio) measures. Glazier et al. 2009 used four features to define walkability, population density, residential density, number of retail and services, and street connectivity [21]. Spoon (2005) identified eleven characteristics of walkability, which included what they defined as “essential”, “encourage”, and “extra” characteristics. Essential characteristics were mixed land use, accessibility/convenience, presence of pedestrian facilities, and high connectivity. Encouraged characteristics were a street pattern, density, aesthetics, presence of parks, plazas and open spaces, and traffic calming/street speeds. The two extra characteristics were street orientation and access to transit [12]. In 2009, Lo conducted a review of the literature examining walkability and identified multiple characteristics and measures that encompassed walkability [11]. These included the presence of continuous and well-maintained sidewalks, universal access characteristics, path directness and street network connectivity, the safety of at-grade crossing treatments, absence of heavy and high-speed traffic, pedestrian separation or buffering from traffic, land-use density, building and land-use diversity or mix, street trees and landscaping, visual interest and a sense of place as defined under local conditions and perceived or actual security [11]. Forsyth (2015) identified nine components of walkability [5]. The first four components are conditions that must be met to achieve walkability. These include environments that are traversable, compact or close, safe, and physically enticing [5]. The next three components exist as a result of walkability. These include environments that are lively and sociable, exercise-inducing and have sustainable transport options [5]. The final two components of walkability are multidimensional and holistic [5]. Despite the interest in green spaces and natural environments [22], few measurement attempts have systematically incorporated assessments of features of the natural landscape (e.g., green canopy, wooded areas, lakes/ponds, parks) [23]. Although research has focused on measuring walkability and summarizing common characteristics that operationalize walkability, a common conceptual definition is still lacking. The objective of this paper was to discuss and propose a conceptual definition of walkability and related concepts.

## Methods

Leaders of the Canadian Urban Environmental Health Research Consortium (CANUE) in partnership with researchers at Memorial University hosted "The Future of Walkability Measures" Workshop in St. John's (Newfoundland, Canada) November 15–17, 2017. Workshop participants were selected based on their expertise in the area of walkability and urban planning and to represent participants at various career stages by the workshop organizers. From across the country, twenty-one leading interdisciplinary researchers and trainees involved in environmental health in Canada participated (See Table 1 for participant list). Workshop participants were informed before the workshop started that the intent of the workshop was to publish a summary of the workshop to contribute to the literature already published. All workshop participants provided verbal consent to participate.

**Table 1:** Workshop Attendee List

Highest Degree	Discipline	Career Stage	Group Name
PhD	Public Health	Senior	Bondar
PhD	Geography	Early Career	Bondar
BSc	Geography	Student	Bondar
MSc	Kinesiology	Student	Bondar
PhD	Statistics	Senior	Handfield
PhD	Urban Planning	Postdoctoral Fellow	Hadfield
PhD	Public Health	Mid-Career	Hadfield
PhD	Urban Planning	Mid-Career	Hadfield
PhD	Geography	Senior	MacLean
PhD	Public Health	Senior	MacLean
PhD	Geography	Postdoctoral Fellow	MacLean
MSc	Computer Science	Student	MacLean
PhD	Urban Planning	Senior	Payette
PhD	Geography	Postdoctoral Fellow	Payette
MD	Medicine	Senior	Payette
PhD	Public Health	Senior	Payette
PhD	Public Health	Early Career	Payette
MSc	Kinesiology	Student	Saint-Jacques
PhD	Public Health	Mid-Career	Saint-Jacques
PhD	Geography	Senior	Saint-Jacques
PhD	Geography	Mid-Career	Saint-Jacques

Workshop participants were divided by corresponding author DF into five interdisciplinary teams which were labelled after Canadian astronauts to stimulate participants to “reach for the sky”. Participants were purposefully placed into teams to ensure that each team had representation from leading researchers in environmental health, practitioners, and students (See Table 1). The workshop was divided into a number of different activities designed to achieve a consensus on a definition of walkability. The consensus method used was a modified Delphi Method where consensus was achieved following multiple rounds of in-person discussions [24]. The consensus approach was used because we recognized the knowledge and experience of our participants. In the first part of the workshop, DF presented an overview of walkability definitions and measures. Subsequently, each interdisciplinary team was tasked with discussing past iterations of concepts and measures associated with walkability, what the term walkability may be lacking, and to develop their conceptual definition of walkability. Teams were given 60 min to discuss and come up with their conceptual definition. Following the small group exchanges, each team was given 10 min to share their definition and justification with the entire group in a plenary session. The group collectively discussed the strengths and limitations of each definition. A moderator kept notes of important discussion points. Following the presentations by each group, all participants engaged in a plenary discussion and working session of 2 h. Throughout the process, disagreements about coding and themes were dealt with thorough discussion and continued iterations. Using a consensus approach (i.e., all participants agreed on the final definition following multiple iterations), the group created a single, consensual conceptual definition of Active Living Environments. Below we provide the definitions that were created by each team and a summary of points raised during the group presentations of their definitions to the full group. Following the individual definitions, we present the final conceptual definition created by all workshop participants.

## Results

Though the original intent was to create a conceptual definition of walkability, the discussion revealed that walkability was too narrow a concept. Below we outline the definitions that were created by each of the teams and provide a brief reflection on each definition, including its potential strengths and limitations.

### Group 1 Definition (Team Roberta Bondar)

Definition of Equitable Active Living Potential, “the emergent property of the physical infrastructure (e.g., transportation networks and pathways, housing, retail) and social conditions (e.g., low crime, affordability, safety) that provides individuals and populations opportunities to ambulate themselves and to recreate in a sustainable manner. These opportunities should be provided for ALL citizens regardless of their sex, age, socioeconomic characteristics, and physical/mental health conditions and abilities.”

The Bondar group proposed a new term "equitable active living potential" to replace walkability. Bondar's team definition was detailed and included three main components: the physical environment, social environment, and the characteristics of the individual using the environment. Borrowing from the language of complexity theory [25], the definition proposes equitable active living potential as an emergent property. This means that the potential effects of active living environments cannot be directly predicted from individual built characteristics and that active living environments are more than a sum of individual components [25]. This definition incorporates a social determinant of health lens and highlights the importance of creating healthy and sustainable environments for all. However, this definition is lengthy and the language may not translate easily to research and policy outside of public health. Beyond ambulating (i.e., walking), this definition also does not make provision for any other type of activity that may be important for health and well-being. This group particularly emphasized that walking and the health benefits of physical activity are important but other health benefits are also important including air pollution, greenness, and road traffic injuries [26, 27].

### Group 2 Definition (Team Chris Hadfield)

Definition of walkability, “a built and natural form which supports safe, convenient, and pleasant access to desired people and places.”

This definition is succinct and easy to understand. The definition included key considerations for walkability such as the need for safety and convenience within the built and natural environments [11, 12], and the social environment [5]. However, the definition did not acknowledge the need for the environment to promote physical activity [4–6] or  improve health.

### Group 3 Definition (Team Steve MacLean)

Definition of walkability, “given the biological need for movement, the total of the features of the natural and built environment that support active living.”

This definition focused on human movement and physical activity. It emphasized that both the natural and built environment play important roles in supporting active living [6]. This definition did not acknowledge the potential influence of the social environment [5, 11] on active living. It also did not take the social determinants of health lens and it did not address equity considerations.

### Group 4 Definition (Team Julie Payette)

Definition of walkability, “a set of environmental characteristics that promote health by encouraging the use of sustainable modes of transportation to access social and personal resources. There are two levels of walkability at the macroscale and microscale that operate differentially on people’s decision making.”

The Payette definition includes a discussion of the influence of environmental characteristics on the use of active transport (cycling, walking, public transport). The definition also suggests that the key outcome of walkability is not the physical activity but rather accessing resources using active transportation. This thinking reflects health promotion language from the 1986 Ottawa Charter for Health Promotion [28], defining health as a resource for everyday life and not an end in itself. This definition is based on the premise that environmental modifications will result in behaviour change, which will lead to improved health outcomes. The macro- and microscales of walkability are not well explained conceptually or operationally in this definition. However, multiple tools have been developed to measure the macro-and microscales of walkability, including the MicroScale Audit Tool for Pedestrians (MAPS), Neighbourhood Environment Walkability Survey (NEWS), Irving Minnesota Inventory (IMI), Pedestrian Environment Index (PEI), the Vancouver Walkability Index (VWI) and the Virtual-STEPS tool [29–34].

### Group 5 Definition (Team David Saint-Jacques)

Definition of walkability, “a measure of the quality of the local built, natural, and social environment for supporting behaviours that promote health and well-being for all.”

The Saint-Jacques group included the built, natural, and social environment in their definition. This definition also highlighted how walkability can influence health and well-being. This group was unique as it emphasized the quality of the environment. This suggests that not only are presence or absence of walkability features required but also the quality of the features may play an important role in supporting active living. The Saint-Jacques definition did not include explicit mention of the promotion of physical activity. The definition provided by this group is quite broad. As a result, the definition is not necessarily linked to active living or physical activity and risks going beyond any other proposed definition of walkability.

#### Conceptual definition

Based on the exchanges held at the workshop, participants unanimously agreed that the term “walkability” should be extended with the broader term Active Living Environments (ALEs). Using ALEs rather than walkability represents one way to acknowledge that those environmental features related to the promotion of physical activity and health are about more than just walking. Based on the definitions used in the literature and the definitions and discussions that emerged from the "Future of Walkability Measures" workshop the need for a more encompassing approach was supported by all. In line with this view, workshop participants discussed and unanimously agreed on the following conceptual definition of Active Living Environments (ALE): the emergent natural, built, and social properties of neighbourhoods that promote physical activity and health and allow for equitable access to health-enhancing resources.

## Discussion

We believe that the term Active Living Environments and the proposed definition redefines previous terms in a way that is accessible to the general public, practitioners, and researchers. Terms such as walkability, Neighbourhood Active Living Potential [6], and Neighbourhood Physical Activity Environments [4] are similar to our new term Active Living Environments (ALE). We also note that the term ALE already has some traction in the literature and has been used in several research papers as a useful alternative to the term walkability [35–39]. In this way, ALE extends previous work, while including the broader definition and conceptualization that is required for knowledge advancement in this area. It is clear that walkability is a popular term that will continue to be used by the media and other organizations, and in some cases (e.g. those explicitly focusing on walking environments) will be appropriate in a research context. Walkability-focused research should continue to advance with the understanding that walkability can be conceptualized as a component of ALEs. The concept of active living environments allows for a broader understanding of multiple types of physical activity beyond walking [8, 9]. The proposed definition encompasses the emergent natural, built, and social properties of neighbourhoods, which all contribute to a neighbourhood’s potential to encourage active living and health [4]. This focus promotes the notion that built, natural, and social characteristics of neighbourhoods are part of large urban areas, that a single aspect of an environment does not define an active living environment, and that the effect of single characteristics is more than the sum of their parts [25]. A limitation with the definition is that the term neighborhood is itself challenging to define and operationalize[40] and does not capture both city or street-level considerations. In their definitions groups tended to use concepts of physical and environmental infrastructure and one group used the concepts micro and macro scale. Using the term neighborhood anchors the definition in the city building process as decisions about features that would improve the active living environment in a city are often made at the neighborhood level.

A focus on physical activity appears pivotal as it is hypothesized to be the primary mechanism through which active living environments promote health. That said, including health in the definition allows for a complementary focus on multiple outcomes including mental health, social connectedness, and safety, among others [5, 11, 12]. An active living environment provides opportunities for people to access health-enhancing resources regardless of the socioeconomic characteristics of individuals or how those characteristics shape urban design [12]. The definition focuses on health enhancing resources. However, areas with many health enhancing resources often have many resources in general, both healthy and unhealthy. The term ALEs better reflect the nature of the concept and includes equity directly within the definition. However, the inclusion of equity was debated during the consensus-building process for the definition, only one group explicitly included equity in their definition. However, the discussion revealed that equity was often implicit in group definitions and should be made explicit because equity is often lost when it is not included explicitly [41]. Of note, the definition does not provide guidance on judging whether an ALE is equitable or not and this will need to be operationalized.

Previously proposed terms such as Neighbourhood Active Living Potential [6] and Neighbourhood Physical Activity Environments [4] are similar to the new concept of Active Living Environments (ALE) in that they both acknowledge the importance of active living within a neighbourhood. These terms align with emerging literature, as researchers are now aware of the importance of the neighbourhood environments and how they influence health. The term Neighbourhood Active Living Potential is limited by not specifically including the word ‘environment’ in its definition and focusing on the difficult-to-define notion of the neighbourhood. The reader is left with the challenging task of defining a neighbourhood [6]. Our proposed definition, similar to Hajna et al., (2017) acknowledges the importance of active living but also clarifies which attributes (physical, natural, and social) create environments [4]. When comparing our proposed definition with that of Gauvin et al., (2005), there are several similarities and differences [6]. NALP is defined as “the aspects of the neighbourhood that regulate the likelihood of active living in individuals and populations” [6]. The mention of the ‘aspects’ of a neighbourhood provides only a vague idea of which factors of the neighbourhood affect the likelihood of active living. In contrast, the proposed definition designates equitable access to health-enhancing resources which can be built, natural, and social features of environments while still encompassing the key aspects of active living in the neighbourhoods where people live. In this way, these three types of environments work simultaneously to promote physical activity and health.

While we believe our conceptual definition of walkability incrementally improves on previous definitions, we acknowledge that our definition was developed by researchers in Canada and may not fully reflect international perspectives. However, this is an important area for future research.

## Conclusion

Creating a new conceptual definition and term to extend the definition of walkability will allow for a consistent understanding and use of the term. This has the potential to increase collaboration and cooperation between the multitudes of researchers who work across disciplines that contribute to building healthy neighbourhoods. Future important steps include creating a reliable and valid set of measurement options across a variety of jurisdictions both within and across countries.

## Data Availability

Meeting notes for the “Future of Walkability Measurement Workshop” will be made available upon request to the corresponding author.

## Abbreviations

ALE: Active Living Environments
CANUE: Canadian Urban Environmental Health Research Consortium
IMI: Irving Minnesota Inventory
MAPS: MicroScale Audit Tool for Pedestrians
NALP: Neighbourhood Active Living Potential
NEWS: Neighbourhood Environment Walkability Survey
PEI: Pedestrian Environment Index
VWI: Vancouver Walkability Index
